# *“It’s Somebody else’s responsibility”* - perceptions of general practitioners, heart failure nurses, care home staff, and residents towards heart failure diagnosis and management for older people in long-term care: a qualitative interview study

**DOI:** 10.1186/1471-2318-13-69

**Published:** 2013-07-05

**Authors:** Helen Close, Helen Hancock, James M Mason, Jerry J Murphy, Ahmet Fuat, Mark de Belder, A Pali S Hungin

**Affiliations:** 1 Durham Clinical Trials Unit, School of Medicine, Pharmacy and Health, Durham University, Queen’s Campus, Wolfson Research Institute, University Boulevard, Stockton-on-Tees TS17 6BH, UK; 2 Department of Cardiology, Darlington Memorial Hospital, County Durham and Darlington NHS Foundation Trust, Hollyhurst Road, Darlington DL3 6HX, UK; 3 Department of Cardiology, The James Cook University Hospital, South Tees Acute Hospitals NHS Foundation Trust, Marton Road, Middlesbrough TS4 3BW, UK

**Keywords:** Heart failure, Quality of care and outcomes, Long-term care, Older people

## Abstract

**Background:**

Older people in care-facilities may be less likely to access gold standard diagnosis and treatment for heart failure (HF) than non residents; little is understood about the factors that influence this variability. This study aimed to examine the experiences and expectations of clinicians, care-facility staff and residents in interpreting suspected symptoms of HF and deciding whether and how to intervene.

**Methods:**

This was a nested qualitative study using in-depth interviews with older residents with a diagnosis of heart failure (n=17), care-facility staff (n=8), HF nurses (n=3) and general practitioners (n=5).

**Results:**

Participants identified a lack of clear lines of responsibility in providing HF care in care-facilities. Many clinical staff expressed negative assumptions about the acceptability and utility of interventions, and inappropriately moderated residents’ access to HF diagnosis and treatment. Care-facility staff and residents welcomed intervention but experienced a lack of opportunity for dialogue about the balance of risks and benefits. Most residents wanted to be involved in healthcare decisions but physical, social and organisational barriers precluded this. An onsite HF service offered a potential solution and proved to be acceptable to residents and care-facility staff.

**Conclusions:**

HF diagnosis and management is of variable quality in long-term care. Conflicting expectations and a lack of co-ordinated responsibility for care, contribute to a culture of benign neglect that excludes the wishes and needs of residents. A greater focus on rights, responsibilities and co-ordination may improve healthcare quality for older people in care.

**Trial registration:**

ISRCTN: ISRCTN19781227

## Background

There is considerable international evidence that the quality of healthcare for long-term care residents is inferior to that for older people living in their own home [1,2]. The healthcare of older people has undergone a major shift from hospital to long-term care facilities [3,4]. Research suggests variable or incomplete assessment and reactive treatment practices, substantial levels of unidentified or misdiagnosed health needs, and a lack of information about the medical and dependency characteristics of older residents [4]. Accurate diagnosis and effective management are challenging as a result of heterogeneity of presentation, unpredictable disease trajectory, cognitive impairment, comorbidities, and polypharmacy [5,6]. Doctors managing the complex medical problems of these often frail older people face difficult trade-offs balancing symptom control, medication burden and quality of life [6]. Complexity may be further compounded by social and residential circumstances and a reticence to intervene unnecessarily. Together these factors may result in an unacceptably high level of acute illness, inappropriate hospital admissions and mortality [1-6].

Heart failure (HF) is responsible for significant morbidity and mortality in older people, placing a substantial burden on the health care system; [7,8] it accounts for 5% of medical admissions (with mean length of inpatient stay 14–16.5 days) [9,10] and is associated with high readmission rates [10]. Recent research suggests a mean prevalence of HF in nursing homes of 20% (range 15–45%) [11-13]. Despite the high prevalence of HF in older people, and the increasing proportion of this population residing in care, [14] there is a lack of research evaluating assessment, diagnosis or care provision in this context. In this setting, acute care often takes precedence over chronic care and prophylactic needs, [15,16] and access to health services may be limited. Older people tend to be investigated and treated less intensely for HF [17]. Evidence suggests older peoples' expectations of treatment are lower as they assume their age is the cause of feeling ill [18]. Older people in care are often physically or mentally frail and may be overlooked or denied health services [17]. Little is known about experiences of health care provision for HF in a population often excluded from research [19]. This qualitative study examines the experiences of HF diagnosis and management from the perspective of residents, care home staff and healthcare professionals.

## Methods

### Recruitment and sampling

The interviews were nested within a larger study (the Heart Failure in Care Homes (HFinCH) study [20-22]) which assessed the prevalence of HF by clinical evaluation and portable echocardiography; and included a randomised controlled trial comparing outcomes for HF patients treated with usual GP care or a tailored, consultant-led management plan delivered by HF nurses (HFN). The GP, HFN, care home staff, and resident interviews were conducted at the end of patient follow-up. The HFinCH study was located in 33 residential and care homes in North East England, within one Primary Care Trust. Care homes were served by a total of 23 urban GP practices (including 93 GPs) which ranged in practice size, representing deprived, affluent, and mixed (both affluent and deprived) populations, and were staffed by a mix of GP partners and salaried GPs. The care homes ranged in size (bed numbers of 17 to 90) with a mix of patients with residential and nursing needs and staffed by a range of staff from qualified, experienced nurses to newly recruited untrained care assistants. Care home staff implemented a variety of HF pathways. The HF nurses work across several urban PCT boundaries and include experienced nurses with specialist qualifications, and more junior staff working towards those qualifications. All residents, aged >65 years recruited to this qualitative study were diagnosed with Left Ventricular Systolic Dysfunction (LVSD) HF during the diagnostic study. Residents had a mix of residential and nursing needs, originated from both affluent and deprived areas, and had a range of co-morbidities consistent with the national picture.

GPs, HF nurses, and care home staff provided written informed consent. Consent was sought directly by HC and HH from residents, or relatives (or consultees) when residents lacked capacity. Resident capacity was determined by the mini mental state examination (MMSE) [23] and abbreviated mental tests score (AMTS) [24]. Written consent included permission to access medical and care facility records.

Purposive samples of residents, care home staff and residents’ general practitioners were interviewed. Participants exposed to either or both trial interventions (‘usual care’ or ‘HF service + usual care’) were included, as were the HFNs involved in delivering the HF service. Recruitment ended when data saturation [25] was reached, i.e. when no new themes emerged as assessed by two independent researchers (HC and HH). Relatives were not formally interviewed, but data on participation rates and rationale for non/participation given on behalf of residents are included.

### Interviews

A transcendental phenomenological [25] methodology was used to describe experiences through the eyes of participants, rather than presenting interpretative representations. Unlike other methodologies such as interpretive (hermeneutic) or social phenomenology, the transcendental methodology requires the researcher to withhold or ‘bracket’ pre-conceptions about the research question [25,26]. Transcendental phenomenology has been used to improve understanding about deeply personal experiences of ill-health such as hearing loss [27], organ transplantation [28], and chronic pain [29]. A recent study demonstrates its utility in exploring the experiences of long term care residents in care [30]. In the current study, it was imperative to allow the voices of residents to emerge undistorted by preconceptions or biases about the potential merits of heart failure care. Alternative phenomenological methodologies (such as hermeneutics) assert that the researcher’s presence shapes the experience under investigation [31]; these were rejected for use in this study on the basis that this inter-subjectivity may inadvertently subjugate the voice of vulnerable older people. In-depth interviews lasting up to one hour with participants were conducted by HC and HH in care facilities and general practices. These examined the care participants currently received (i.e. ‘what' they experienced) and the way in which care was provided (i.e. ‘how' they experienced care in terms of the conditions, situations and context) from the perspective of residents, care home staff and clinicians. Interviews with residents were conducted in the residents’ own rooms, those with carers and health professionals were held in office premises. A short interview schedule was used which encompassed experiences of HF diagnosis and management and general views on the healthcare of older people in care. Interview questions were open-ended.

### Ethical approval

The study complies with the 1975 Declaration of Helsinki, the study received prior local research management and governance and national ethics approvals from Leeds West REC (Reference: 08/H1307/96). All participants provided written informed consent prior to participation.

### Data analysis

Interviews were tape-recorded and transcribed verbatim. Transcripts were read and analysed independently by HC and HH using thematic analysis [25] to explore the ‘lived experience' of participants. During the initial stage of analysis, each analyst agreed on a list of preliminary codes, which were added to and refined as coding progressed. Codes were grouped into categories and from these a set of themes emerged which characterised information within categories. Emergent themes were tested using diverse accounts within cases and between cases, in order to challenge the integrity of the boundaries of themes. Recruitment data were analysed to determine the number of relatives who declined participation on behalf of a resident and their reasons, where offered. Direct quotes are shown in italics below with the care received in brackets (usual care =control, HF service + usual care =intervention).

## Results

Of 28 residents with LVSD, we recruited all 17 with capacity to consent (routine care: 8, HF service: 9), 8 care home staff (routine care: 4, HF service: 2, both:2), 5 GPs (routine care: 2, HF service: 2, both:1) and 3 HFSNs (routine care: 1, HF service: 2) (Table 1). From the interviews, themes emerged that may prevent residents, care home staff or clinicians from acting on possible symptoms of HF. These fell into three main categories; challenges concerning the organisation of healthcare for HF, the variable quality of care in care homes, and opposing expectations about healthcare for older people. The overarching theme was the lack of clear lines of responsibility for healthcare and for heart failure in particular.

**Table 1:** Characteristics of interview participants

**Characteristics**	**Interviews**	**Prevalence study**
**Residents in care**	17	399
Mean (SD) age, (range)	85.3 (5.1, 73–94)	84.2 (7.2, 65–100)
Male	7 (41%)	105 (26.3%)
White British	17 (100%)	393 (98.5%)
White European	0 (0%)	6 (1.5%)
*Care home type*		
Nursing	4 (24%)	120 (30%)
Residential	12 (71%)	263 (66%)
Dementia	1 (6%)	16 (4%)
**General practitioners**	5	93
**Care home staff**	8	31
**HF specialist nurses**	3	3

### Organisation of health care for heart failure

The majority of participants referred to barriers which limited access to generic health care, as well as specifically for symptoms suggesting HF. Access was mediated by a complex combination of individual, organisational and attitudinal barriers, which revealed the disparate nature of processes and goals within health services and care facilities. Standard referral for an echocardiogram involved a hospital visit that was often not welcomed by residents or staff for practical reasons.

Care home manager: “They get themselves so worked up if they're going to hospital because …. they can be on an ambulance an hour and a half before they get there, then they can be sat an hour and a half before they get seen … so that by the time they get back they're frozen stiff, they're hungry, they've had nothing to eat or drink all day and they're as stressed as hell …” (CHM1, control and intervention)

GPs reported a reluctance to refer for diagnosis or specialist treatment for reasons including comorbidity, immobility, and access difficulties, alongside recognition that older people may be more willing to tolerate symptoms than younger people. Reluctance to refer for more aggressive treatment appeared grounded in a concern about the balance of risks and benefits, and ultimately, the well-being of the resident. There were also strong doubts that a diagnosis would positively influence symptom management. GPs reported a sense of anxiety about the risks of treatment for older people and conceptualised the healthcare challenges for this group firmly within the bio-physical experience of symptoms and side effects. There were conflicting views about the relative importance of symptoms and signs suggesting HF.

Care Home Manager: “We’ve had to say … from a nursing point of view, we’re not challenging you but we’re not quite sure about this especially if the input is low and their ankles are swollen.” (CHM5, control and intervention)

Residents randomised to the HF service valued the additional visits, and welcomed both the changes made to their treatment as well as the opportunity to participate in decisions about their care. Some reflected on the positive changes they noticed to their levels of oedema and mobility since being treated:

Resident: “Yes, well, for instance I couldn't wear these shoes before. I had to get somebody to get me those canvas things that were comfortable and bigger, but they’re too big now.” (R4, intervention)

Residents randomised to usual care expressed frustration at the perceived lack of regular visits from healthcare professionals and demonstrated a tacit acceptance that symptoms and ill health are an unavoidable part of the aging process. These residents expressed a reluctance to learn or understand more about their condition or about their treatment; their verbal and non-verbal language suggested withdrawal and resignation about their discomfort and frailty:

Resident: “Well I keep taking the tablets at the end of the day but I haven't a clue what they're for.” (R7, control)

Several residents randomised to usual care wanted to know more about their health and their treatment plans but felt unable to ask questions, partly because of their respect for clinical work and the precedence of the doctor’s view over their own thoughts and wishes, and partly because of the organisation of care:

Resident: “You've got to go through a second channel to get to the GP here. I've got to inform the nurse, … at home, I just used to get on the phone and make an appointment and that was it.” (R8, intervention)

Any improvements in HF symptoms were welcomed and were largely expressed in relation to social and physical function.

Resident: “I mean I go to the hairdressers once a fortnight now … I got that I wasn’t able to go at all … … and even the hairdresser says "you've improved a lot". (R11, intervention)

Some referred to the difficulties experienced in relation to diuretics though accepted the fine balance between effective treatment and side effects:

Resident: *“I just hope that whatever is going to be done will make me feel a bit better.” (R3, intervention)*

### Quality of care

GPs and HFNs all expressed frustration about the lack of continuity of staff in care facilities, making it difficult to access in-depth knowledge of the needs of residents. The majority of staff were untrained and relied on external clinicians to guide decisions about healthcare; nursing homes had at least one qualified nurse per shift. Clinicians all commented on the variability in quality of care between and within the facilities, which they attributed to the presence or absence of qualified nurses:

HFN: “It's so variable … you know instantly when you go to certain care homes that you're going to do well there or the patient’s not going to do well.” (HFN1, control and intervention)

The implications of the lack of qualified staff were not always immediately apparent but had potentially serious longer-term consequences for the health of residents:

Care Home Manager: “When I was a ward sister the majority of clients we would get were from a care home - we were dealing with dehydration, that was all that was wrong with them but it then precipitated to other problems.” (CHM4, control and intervention)

Care home staff and clinicians expressed a lack of ownership or oversight of the patient’s care and wellbeing:

HFN: “It probably sounds silly but, you know, you do feel like it's somebody else's responsibility and you're dipping into it really.” (HFN2, control and intervention)

Other clinicians delegated responsibility for care to other staff:

GP: “There’s a lady who’s diagnosed, she’s in a residential home… I’ve got her on 10mg ramipril but I’ve never seen her… I’ve had health care assistants titrating up the ramipril … I’ve done it by telephone … I think that’s appropriate.” (GP5, control)

Most clinicians felt that when comparing types of home, the residents actually varied very little in the types and severity of their needs. However, the interface with NHS services was different for nursing homes and residential care homes The HFNs noted particular challenges in getting prescriptions dispensed and reaching the patient, while care facility staff were frustrated that many of the residents did not appear to be offered routine medication checks:

Care Home Manager: “When that medication should have been stopped or it was short term and you find out they’ve been on it for 5 months because nobody’s picked up on it … that’s a big problem for us.” (CHM1, control and intervention)

### Opposing expectations about healthcare for older people

Through the process of obtaining consent for the trial it became apparent that the views of relatives and GPs were polarised; many welcomed the study and its opportunities for improved care, however, a substantial number rejected the opportunity. Relatives who took the latter view responded negatively to invitations to participate on the basis that intervention would extend length of life without corresponding improvements to quality. Some GPs saw little merit in changing the current system for older people referring to a lack of evidence for any change:

GP: “I am not familiar with the evidence of the benefit of treating heart failure in older, less ambulant patients.” (GP3, control and intervention)

However, the majority of residents were very keen to participate in the trial:

Resident: “I think I felt a bit more specifically looked after …they are mainly carers that are looking after you, not nurses, it's nice that somebody cares about this particular thing, and knows about it.” (R8, intervention)

Some care facility staff expressed a sense of distrust that treatment decisions were made solely on a risk/benefit assessment; instead they perceived that older people in care were a low priority to health service providers. Information provided by care facility managers, augmented by note review, indicated a lack of HF specific care pathways or multi-disciplinary team meetings in any of the homes:

*GPs: “Obviously 50 years olds and 60 year olds, you’ll get them on as much as possible as long as they don’t have any side effects but I think with the elderly you don’t push them as much as I would with a younger person.”* (GP2, control and intervention)

Care Home Manager: “Unfortunately, a few people have the attitude 'well they're old so what's the point' but the point is, even though they're old, they still need a quality of life and just because they're old doesn’t mean that they should be neglected.” (CHM1, control and intervention)

## Discussion

This is the first study to describe the lack of clear lines of responsibility for the diagnosis and treatment of heart failure (HF) in older people in long-term care. Clinicians and care home staff both believed that decision making, care delivery, and its co-ordination were the responsibility of others; both groups recognised the status quo as untenable, but appeared unable or unwilling to develop alternatives. This situation perpetuated a culture of benign neglect which excluded the wishes and needs of residents and contributed to poor quality heart failure management. Clinical staff and relatives tended to base decisions about HF intervention on assumptions that these may be challenging or unwelcomed by residents. These decisions either countered the wishes of residents or were not discussed with them. Given the opportunity, most residents wanted to be involved in decisions about their health, which included HF diagnosis and treatment. Despite preconceptions that HF diagnosis and management would be unwelcome or difficult, the onsite heart failure service offered during the trial overcame many personal and organisational barriers. This consultant-led service was highly acceptable to residents, care home staff, and heart failure nurses, although some relatives and general practitioners remained unconvinced.

This study encompassed a wide range of long-term care organisations and healthcare models without onsite health care facilities or access to resident medical records. Alternative models which include onsite, non-specialist, un-integrated services (e.g. in the US and the Netherlands) have not adequately addressed unacceptable variability in heart failure care [32-34]. In this study the consultant-led on-site heart failure service successfully mediated between clinicians, carers, relatives and residents, thus providing a co-ordinated model care. This supports recent research which highlights the importance of the heart failure nurse in the co-ordination of care [35]. This has important implications for the organisation of generic healthcare for older residents in long-term care.

Some narratives provided by patients, carers, and clinicians were challenging and prompt the need for a broader social debate about appropriate healthcare for this population. The refusal of diagnostic tests by relatives on the basis that treatment might extend life was surprising. Judgements about the value of one’s life should be sought from each individual, however this was not always possible to achieve. Two thirds of residents in our prevalence study were cognitively impaired, meaning that relatives were consulted about participation. For research ethics reasons a relative’s view was accepted even when it countered the view of the resident and even when the resident had capacity to consent. Some relatives believed that the study would prolong the life of residents without achieving improved control of the symptoms of HF. The view by some participants that care for heart failure is inappropriate for this group may explain the unwillingness or inability of residents to have a voice in decision-making despite their desire to do so. Residents saw themselves as passive recipients of care and viewed themselves as ‘only one of many’. This is consistent with recent international research with this population [36,37]. This body of research mirrors the low expectations and sense of apathy experienced by older people in our study but also highlights the fact that older people are willing and able to participate in healthcare decisions given the appropriate investment in skills, resources and time [37-39]. Similarly, we found residents were keen to participate in the HFinCH study, which provided the opportunity for diagnosis and specialist management of heart failure, as well as to recount their experiences of the study and their care in general. Decisions about clinical management or participation in research may often be polarized between the active engagement of older people and the outright refusal of relatives, carers or those with power of attorney. Conflicting opinions between older people and their well-meaning relatives may be common and problematic both for research recruitment and for clinical care. Resolving these conflicts may involve a paternalistic decision to uphold the relatives’ viewpoint (not rocking the boat), but this has moral and ethical implications requiring a wider debate about the role and appropriateness of care for older people.

### Study limitations

The study was designed to elicit the experiences of residents; we therefore did not formally interview relatives to ascertain their views, although informal conversations were revealing. Given the role of relatives as gatekeepers of healthcare, this is a limitation of this study and is an important avenue for future research. A second potential limitation of the study was that we did not attempt to elicit interview data from cognitively impaired residents due to ethical concerns; our experience suggests that, given time and encouragement, this group were often willing and able to express views and beliefs about care. The use of transcendental phenomenology had both strengths and weaknesses in this study. Previous research has demonstrated its utility in producing highly descriptive data to illuminate personal experiences of health and illness [27,31]. As such it is an important tool to encompass experiential elements of healthcare that may otherwise be disregarded. However, this approach depends on the ability of research participants to produce meaningful descriptive data, a task which proved challenging for some of the residents in this study. Some residents either did not or could not engage in meaningful discussions about their experiences of health and illness and often preferred to discuss their families or previous major life events. The research was conducted predominantly with working class older people in the North of England; it is possible to suggest a cultural reluctance to engage in critique about healthcare, which contrasted with the ability and willingness of residents to talk at length about other personal issues. It is possible that alternative methodologies using interactive methods might in future elicit further in depth data with this population.

## Conclusions

Some clinicians expressed the view that care for heart failure was inappropriate for residents in long-term care, which may explain the unwillingness or inability of residents to influence decision-making despite their desire to do so. Residents saw themselves as passive recipients of care and consistent with previous research [19,36,37] viewed themselves as ‘only one of many’. However study findings demonstrate that, given the opportunity, appropriate investment in skills, resources and time, older people are willing and able to participate in healthcare decisions [36,38] and research. Decisions about such participation may often be polarized between the active engagement of older people and the outright refusal of relatives, carers or those with power of attorney. Conflicting opinions between older people, their well-meaning relatives and clinicians may be common and problematic for research and for clinical care. These conflicts may currently be resolved by paternalism, disregarding the resident’s view, but this has moral and ethical implications requiring a wider debate about the role and appropriateness of care for older people.

## Competing interests

The authors declare that there are no competing interests.

## Authors’ contributions

HC led data collection, participated in data analysis, interpretation and writing this manuscript. HH co-designed the study, participated in data collection, data analysis, interpretation and writing this manuscript. JMM co-designed the study, participated in data interpretation and writing this manuscript. JJM participated in data interpretation and writing this manuscript. AF participated in data interpretation and writing this manuscript. MdeB participated in data interpretation and writing this manuscript. APSH devised the research question, co-designed the study, participated in data interpretation and writing this manuscript. All authors read and approved the final manuscript.

## Pre-publication history

The pre-publication history for this paper can be accessed here:

http://www.biomedcentral.com/1471-2318/13/69/prepub
